# Age-dependent hypopharyngeal gland size and protein content of stingless bee workers, *Tetragonula pagdeni*

**DOI:** 10.1371/journal.pone.0308950

**Published:** 2024-08-16

**Authors:** Lars Straub, Tanatip Sittisorn, Jinatchaya Butdee, Woranika Promsart, Athitta Rueangwong, Domenic Camenzind, Jakkrawut Maitip

**Affiliations:** 1 Institute of Bee Health, Vetsuisse Faculty, University of Bern, Bern, Switzerland; 2 Faculty of Science, Energy and Environment, King Mongkut’s University of Technology North Bangkok, Rayong, Thailand; 3 Centre for Ecology, Evolution, and Behaviour, Department of Biological Sciences, Royal Holloway University of London, Egham, United Kingdom; Gomal University, PAKISTAN

## Abstract

Eusocial insects, such as stingless bees (Meliponini), depend on division of labour, overlapping generations, and collaborative brood care to ensure the functionality and success of their colony. Female workers transition through a range of age-specific tasks during their lifespan (i.e., age-polyethism) and play a central role in the success of a colony. These age-specific tasks (e.g., brood care or foraging) often closely coincide with key physiological changes necessary to ensure optimal performance. However, our understanding of how nutrition, age, and polyethism may affect the development of such physiological traits in stingless bees remains limited. Here we show that pollen consumption and age-polyethism govern hypopharyngeal gland (HPG) acini size and protein content in *Tetragonula pagdeni*. By conducting a controlled laboratory experiment we monitored the effect of pollen consumption on worker bee survival as well as assessed how a pollen diet and age affected their HPG acini width and protein content. Further, we sampled nurses and foragers from field colonies to measure the effect of age-polyethism on HPG acini width. We found that pollen consumption enhanced survival and led to increased HPG acini width and protein content and that HPG acini were as expected largest in nurse bees. Our findings highlight the beneficial effects of an adequate diet for physiological development and health in stingless bees and reveal that age-polyethism is the key factor governing HPG size in worker bees. As HPGs are imperative for collaborative brood care—an essential component of eusociality—the data provide a foundation for future studies to investigate the impact of potential environmental stressors on a critical physiological trait in stingless bees which may serve as a proxy to understand the effects at the colony level.

## Introduction

Stingless bees are amongst the most common bees across the subtropical and tropical regions of the world [1]. Over 500 species have been described of which 57 species from 10 genera are native to Asia [2]. Besides playing a key role as pollinators in natural and anthropogenic landscapes (e.g., agricultural or urban habitats) [3], stingless bees have a long tradition of being managed to produce honey and wax (i.e., cerumen) for personal and commercial purposes [4]. Yet, despite their ecological and social relevance, stingless bees remain understudied when compared to other bees (e.g., honey bees and bumble bees), in particular, as model study systems to better understand fundamental aspects of eusociality or physiology.

As highly eusocial insects, stingless bees live in colonies that consist of several thousands of individuals [4]. Their colonies are shaped by the three pillars of eusociality–(reproductive) division of labour, overlapping generations and collaborative brood care [5]. The female worker caste lays the foundation of a successful colony as they exhibit a range of tasks including foraging, thermoregulation and brood care which are essential for colony functionality and fitness [6]. In stingless bees, the division of labour is age-related (i.e., temporal polyethism), whereby workers transition through a set of behavioural changes over the course of their life. The average lifespan of a stingless bee worker varies and can range anywhere between 30–67 days depending on the species [4]. While precise data on age-related task behaviour is scarce, younger workers are known to be commonly found within the nest performing inhive tasks (e.g., cell building and brood care), whereas older workers will typically conduct riskier tasks outside the nest (e.g., guarding and foraging) [7]. More specifically, nursing behaviour in stingless bees is typically displayed between the ages of 13–18 days [8]; however, is known to be displayed even shortly after worker emergence (e.g., *Tetragonula* spp. [9]). Temporal polyethism not only influences the behaviour but also the physiological development of individual bees. For example, brood attending workers (i.e., nurses) will have highly developed hypopharyngeal glands (HPGs) that are larger and have a higher protein content when compared to older workers (e.g., foragers). The HPGs are pair of secretory organs found in the head of the workers and are composed of several acini which synthesize a proteinaceous substance that is fed to the developing larvae [10]. Once the workers become older and progress from brood care to other tasks, the HPGs degenerate and become smaller in size and have lower protein content (e.g., [10–12]). Therefore, the physiological status of a worker can be used as a proxy to estimate the age as well as task it may currently be performing. An additional factor that affects the health and physiological development of a bee is a balanced diet [13].

Pollen, the primary source of dietary proteins and lipids, is particularly important for longevity [14], immunocompetence [13] as well as gland and organ development [15, 16]. Dietary demands of workers can vary depending on their age and tasks performed within the colony. For instance, nurse bees tend to have a protein-rich diet which is required for optimal development of HPGs and the synthesis of the protein rich larval food [16]. Foragers in contrast, tend to have a carbohydrate-dominated diet which is necessary to accomplish energetically demanding foraging flights. However, our knowledge on how temporal polyethism and nutrition affects physiology in bees derives mostly from studies using honey bees. Far less is known for stingless bees, in particular Asian species, as most existing studies used South American species (e.g., [7, 17–20]). Additional baseline data on physiological traits in Asian stingless bees appears crucial to better understand their biology and needs which would enable more informed conservation efforts as well as improved meliponiculture (i.e., stingless beekeeping) practices.

In Thailand, 33 stingless bee species can be found [21, 22], amongst which *Tetragonula pagdeni* is one of the most common species to be used for meliponiculture [23]. Given they are easily managed and readily available for scientific purposes, this species represents an ideal model system to fill current knowledge gaps on how diet and temporal polyethism can affect adult stingless bee physiology (i.e., HPG acini width and protein content) and longevity (i.e., survival). We accomplished this by conducting a controlled laboratory experiment in which we monitored survival and assessed HPG acini size and protein content at four different time points (i.e., days 0, 3, 10, and 17) that reflect the typical nursing age of stingless bees [8, 9].In addition, we sampled nurse and forager bees from colonies to measure the effect of age-polyethism on HPG acini size. Considering previous studies (e.g., [16, 18, 24, 25], we hypothesized that pollen-consumption will have a significant positive effect on gland development and survival, and that nurse bees will have significantly larger acini when compared to foragers.

## Materials and methods

### Experimental design and treatment exposure

Between March and June 2023, three local queenright colonies of *Tetragonula pagdeni* were used at the King Mongkut’s University of Technology North Bangkok, Rayong, Thailand. Based on visual inspections, all colonies were free of parasites and contained sufficient food stores (i.e., pollen and honey) [26]. To obtain newly emerged bees of the same age cohort, brood patches were gently removed from each colony, immediately placed in plastic containers [500 cm^3^], and incubated under complete darkness at 28 ± 2 °C with 70 ± 10% relative humidity (RH), thereby simulating natural colony conditions [27, 28]. Upon emergence, each worker was visually examined for physical abnormalities or clinical symptoms of disease [4, 29] before being randomly allocated to standard hoarding cages ([200 cm^3^]; [30]).

**Fig 1:**
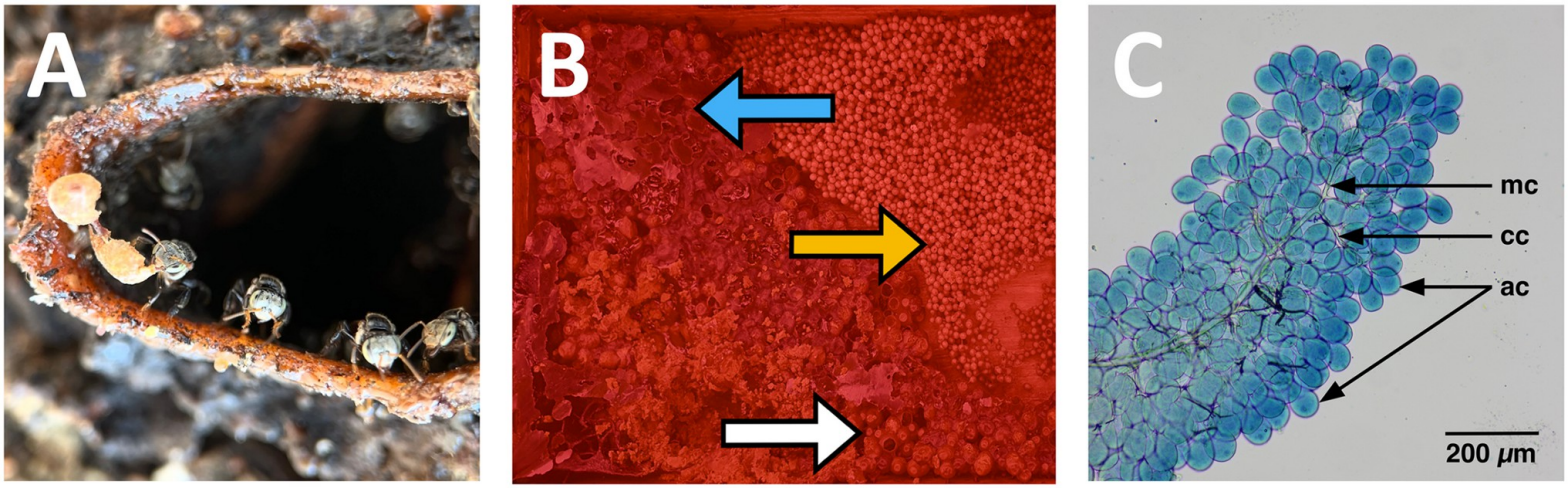
*Tetragonula pagdeni* adult bees, colony and hypopharyngeal glands (HPGs). **A)** Adult workers guarding the nest entrance, removing waste from as well as forages leaving the hive. **B)** Colony under red light at night to enable the sampling of nurse bees (blue arrow indicating honey pots; yellow arrow indicating brood; white arrow indicating pollen pots). **C)** Hypopharyngeal gland acini under a light microscope (**mc**: main canal; **cc**: canal cells; **ac**: acini).

In total, 12 hoarding cages were filled with 30 individuals each, whereby 10 bees were added from each colony to account for possible genetic variation for the tested traits (N = 360 bees total). There were six cages (i.e., replicates) for both pollen-fed treatment and negative control. All cages contained a 5 ml syringe providing 50% [w/v] sucrose solution *ad libitum* to provide sufficient carbohydrates [16]. The treatment cages contained an additional 2.5 ml Eppendorf feeder providing *ad libitum* honey bee corbicular pollen as a protein source [16], whereas controls had no pollen-feeder. According to the commercial pollen provided (Chiangmai Healthy Product Co., Ltd., Chiang Mai, Thailand), the pollen was almost exclusively *Mimosa pudica* (L) and the pollen diet could thus be considered monofloral. *Mimosa pudica*, commonly known as the sensitive plant, is widely distributed in Thailand and its pollen is rich in protein (i.e., 38.71% w/w) as well as having high levels of vitamins and minerals [31]. It is thus an attractive nutritional source for various bee species across Thailand [32]. To limit microbial interference in the food, syringes and feeders were replaced every four days [33]. Within our experiment, the quantification of pollen consumption was not feasible, as the bees had the tendency to remove and spread pollen through the entire cage, as observed in a previous study [34]. Survival was monitored until the experiment was terminated on day 17. To have positive controls, HPG development was assessed under field conditions (i.e., optimal nutrition and brood presence), using 10 nurses and 10 foragers collected from the same three colonies. Subsequently, at least three bees per colony were used to account for potential genetic variability in HPG acini size. To foster that the bees were conducting the appropriate task, foraging bees with pollen were sampled directly from the entrance of the colony upon returning to their colony during the day or directly from the brood patch (i.e., nurses) at night using a red-light torch to minimize colony disturbance (Fig 1A & 1B). Individuals were caught using a brush and gently transferred to a hoarding cage [200 cm^3^]. Lastly, to determine the baseline HPG acini width and protein content, 16 newly emerged bees from the laboratory kept brood patch were assessed.

### Hypopharyngeal gland acini width and protein content analyses

The HPG acini width [μm] and protein content [μg mL^- 1^] were assessed following published standard protocols [12, 35]. To estimate HPG development over time, the HPG acini width (Fig 1C) and protein content were assessed on day 0 (i.e., baseline) and after three, 10, and 17 days. We chose these timepoints as acini size and protein content are known to reach their peak development when workers are typically attending brood between days 13–17 in most stingless bees [4]. For the baseline bees, 10 newly emerged bees from the brood patch were used. On days three, 10 and 17, five randomly selected individuals per treatment were removed across the six cages: resulting in a total of 15 replicates. To account for potential variation, the width of 30 arbitrarily selected acini were measured per specimen [35]. In addition, the acini width was measured for 10 foragers and 10 nurse bees using the same methods as above. The HPG protein content was also measured on days 0 (i.e., baseline), three, 10, and 17. To assess the baseline protein content, an additional six newly emerged bees were selected from the brood patch, and three replicates per treatment and time point (i.e., day three, 10, and 17): resulting in nine bees per treatment.

### Data analyses

All statistical tests were performed in STATA16 [36]. All outcome variables (i.e., mortality [%], HPG acini width [μm], and HPG protein content [μg mL^- 1^]) were tested for normality by visually inspecting quantile-quantile plots as well as using the Shapiro–Wilk’s test and the Levene’s test for homogeneity of variances. Survival data were analysed using Kaplan–Meier survival statistics with the Logrank test (i.e., Mantel–Haenszel test), whereas the survival rate on day 17 (i.e., termination) was tested using binary logistic regression using a standard normal *z*-statistics (i.e., Wald’s test). To account for individuals that were removed from the survival analyses to assess HPG traits, we right censored these individuals as their survival time was considered ‘incomplete’ [37]. Acini width and protein content were both normally distributed (Shaprio-Wilk’s test for normality P > 0.05). To evaluate a potential connection between explanatory variables (i.e., treatment or age) and dependent variables (i.e., HPG acini width and protein content), linear regression mixed-effects models (LMMs) were applied. Generalized linear (regression) models (GLMs) with random intercept were fitted using the STATA function *glm*, wherein bees were considered independent units, ‘treatment’ (i.e., baseline, control, or pollen-fed), ‘age’ (i.e., day three, 10 and 17), and the interaction (i.e., *treatment##age*) were included as the explanatory (fixed) terms, and whenever applicable the random effect ‘cage’ was incorporated. Best fit models were chosen by comparing every multilevel model with its single-level model counterpart using both a likelihood ration (LR) test as well as the Akaike information criterion (AIC) using the function *lrtest* and *estat ic*, respectively. For both HPG variables, if one of the fixed terms revealed significant, a post-hoc test was performed by using a Bonferroni multiple pairwise comparisons test (*bmct*), obtained by using the function *mcompare (bonferroni)*. Whenever appropriate, the means ± the standard error (SE) are given in the text and are further provided in S1 and S2 Tables in S1 File. All figures were created using NCSS v.12 [38].

## Results

### Survival

**Fig 2:**
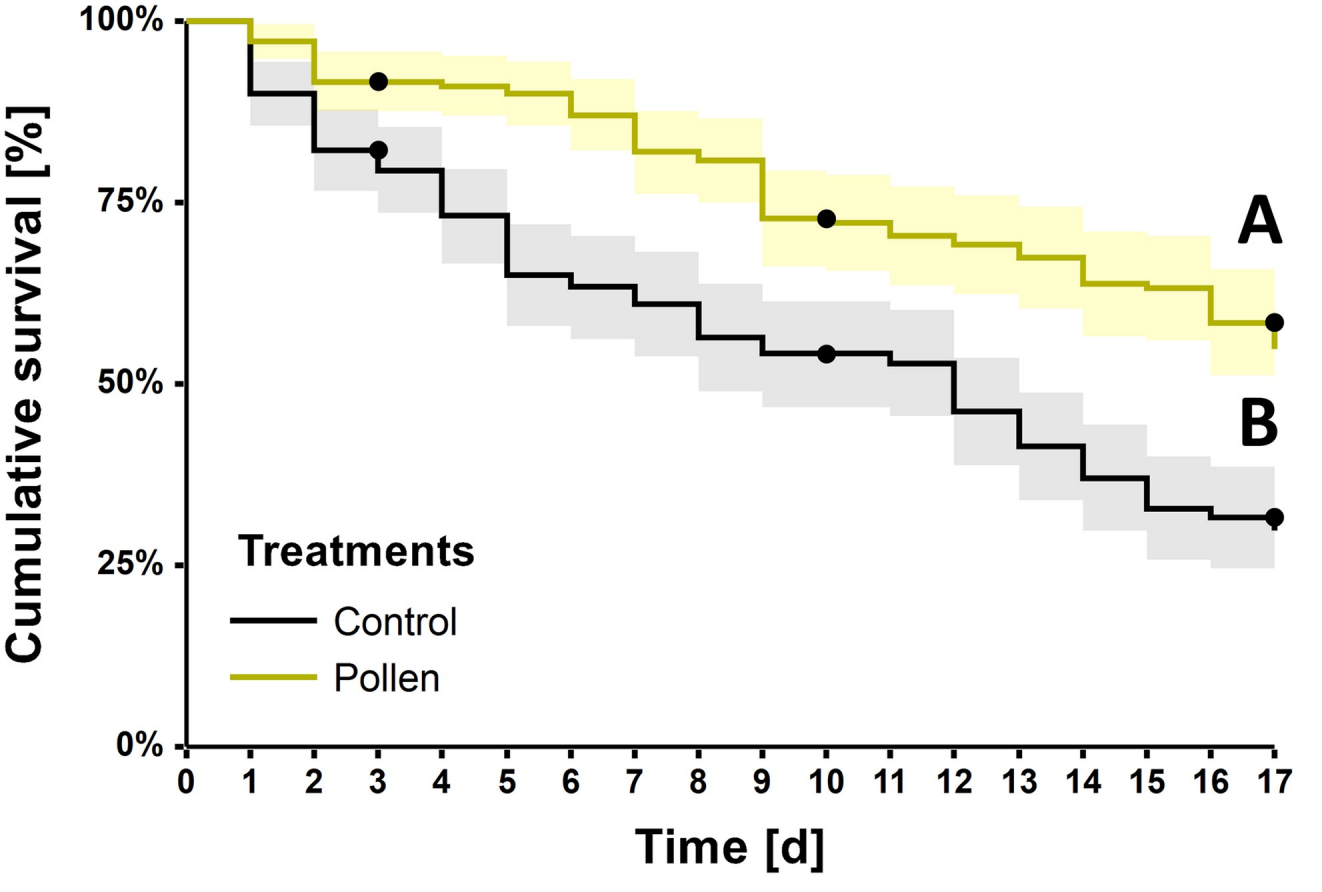
*Tetragonula pagdeni* adult worker cumulative survival curves (Kaplan-Meier). A comparison of survival between pollen-fed workers (*N* = 180) and controls (*N* = 180) revealed a significant difference between treatment groups (*Logrank*, *P* < 0.05), which is indicated by the letters A and B. The solid black dots indicate points of censorship (i.e., days 3, 10 and 17), where a subsample of individuals were removed from cages to assess either hypopharyngeal gland acini size or protein content. Shaded areas surrounding the survival curves represent the 95% confidence intervals.

Pollen-fed bees revealed a significantly increased survival (*Logrank*; χ^2^ = 26.8, *d*.*f*. = 1, *P* < 0.001), where mortality after 17 days was 45% and 70% for pollen-fed and control treatments, respectively (Fig 2).

### Hypopharyngeal gland acini width

**Fig 3:**
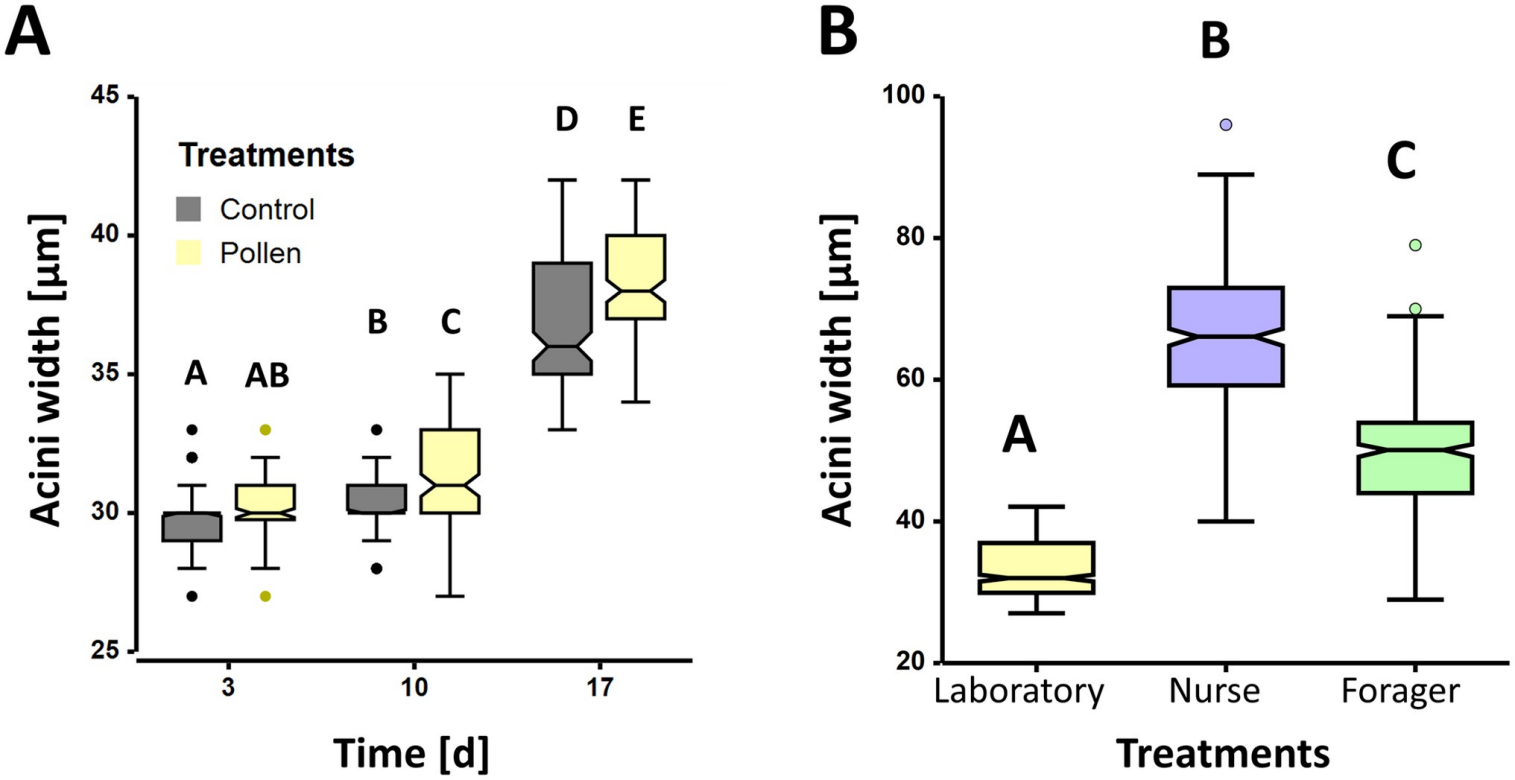
*Tetragonula pagdeni* hypopharyngeal gland (HPG) acini width in adult workers. **A)** Comparison of the acini width in workers at three different time points (i.e., days 3, 10 and 17). With age the acini width significantly increased (*glm*; *P* < 0.05), wherein pollen consumption had a significant positive effect on acini size (*glm*; *P’s* < 0.05). **B)** A comparison of the acini width in pollen-fed laboratory kept workers aged 3, 10 or 17 days (i.e., ‘Laboratory) and workers taken directly from field kept hives found on brood (i.e., ‘Nurse’) or when returning from foraging with pollen (i.e., ‘Forager’). Acini width in worker bees from hives were significantly larger when compared to the acini in laboratory kept pollen-fed bees (*bmct*; *P* < 0.05), wherein the nurses had the largest acini (*bmct*; *P* < 0.05). All box plots show the interquartile range (box), the median (black line within box), the data range (horizontal black lines from box) and outliers (coloured dots). A significant difference between treatment groups is indicated by alphabetical letters.

Baseline (28.55 ± 0.13 [μm]) acini width was significantly smaller when compared to laboratory caged individuals (*glm*; *z* = -18.32, *P* < 0.001). For the laboratory caged bees, both treatment and age had a significant positive effect on acini width (*glm*; both *z*’s > 2.18, *P*’s < 0.03). Likewise, there was a significant interaction between the two variables (*glm*; *z* = 2.39, *P* = 0.017), resulting in a positive linear correlation between acini width and time across both treatments. Irrespective of the treatment, HPG protein content increased with age by 5%, 8% and 32% on days three, 10 and 17, respectively, in comparison to the baseline data. The increase in acini width between days 10 and 17 being ~22%. When comparing pollen-fed bees with controls, no significant difference in acini width was revealed between bees from the pollen-fed (30.2 ± 0.08 [μm]) and control (29.8 ± 0.09 [μm]) treatments on day three (*bmct*; *z* = 0.8, *P* = 1.0; mean ± S.E. [μm]). However, a treatment effect was observed on day 10 (*bmct*; z > 2.98, *P* < 0.043; Fig 3A), where pollen-fed acini (31.3 ± 0.20 [μm]) were significantly larger than the controls (30.3 ± 0.09 [μm]). This effect was also revealed on day 17 (*bmct*; *z* > 36.1, *P* < 0.001; Fig 3A), where comparing pollen-fed acini (38.2 ± 0.15 [μm]) with larger than control acini (36.9 ± 0.14 [μm]). Subsequently, compared to controls the pollen fed HPG acini were 0.1%, 3.3%, and 3.5% wider on days three, 10 and 17, respectively.

When comparing the acini width across positive control field nurses (65.9 ± 0.56 [μm]) and foragers (49.6 ± 0.44 [μm]) with pooled laboratory pollen-fed bees aged three, 10 and 17 days (33.2 ± 0.13 [μm]), significant differences were revealed amongst all groups (*bmct*, all *z*’s > 28.2, all *P*’s < 0.001; Fig 3B). Nurses revealed the largest acini, reflecting a 33% and 98% increase when compared to both positive control field foragers and laboratory treatments, respectively.

### Hypopharyngeal gland protein content

**Fig 4:**
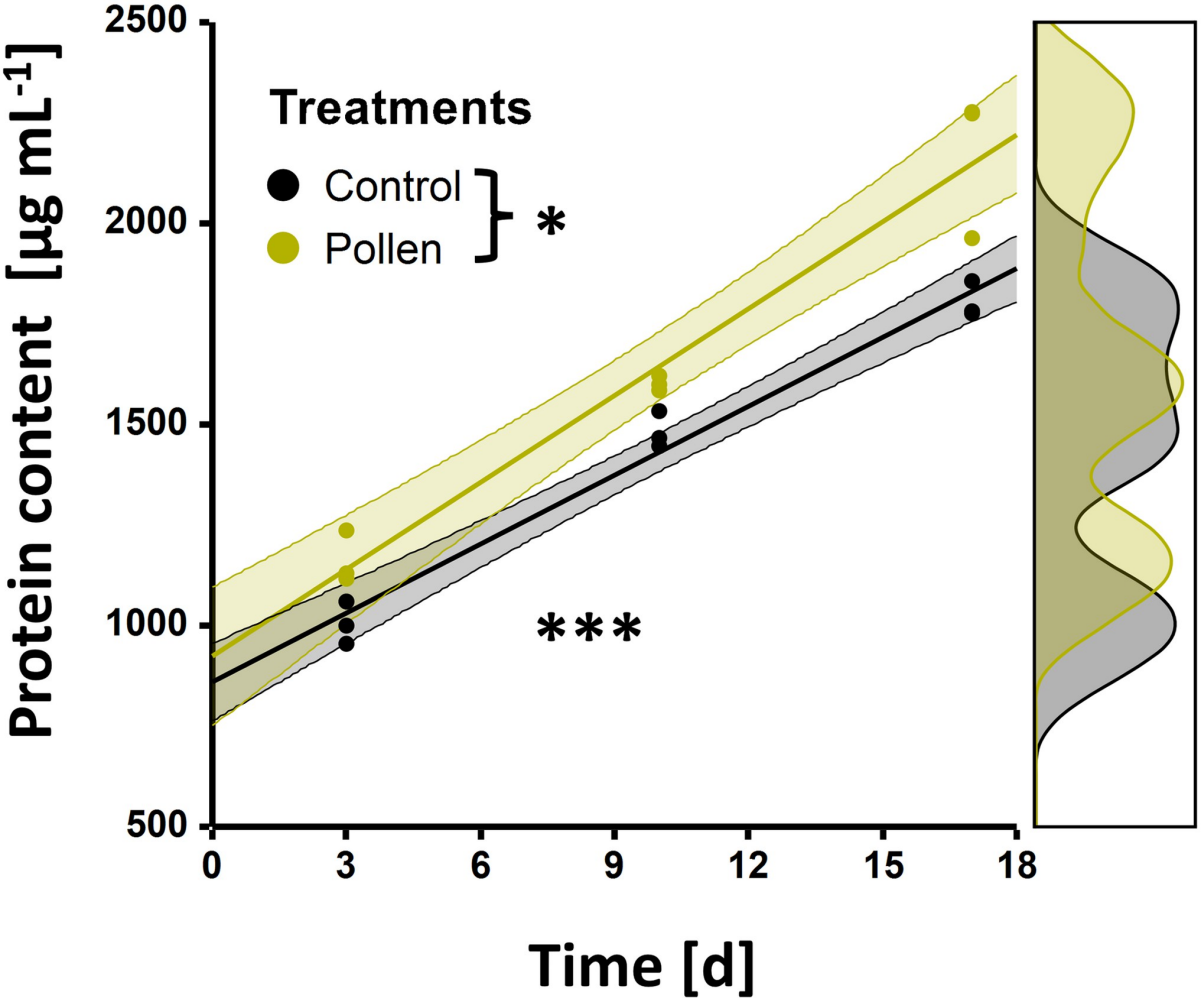
*Tetragonula pagdeni* hypopharyngeal gland (HPG) protein content in adult workers. For each treatment (control = black; pollen-fed = yellow), HPG protein content across time is displayed. A significant positive correlation was observed for protein content and age and a significant difference between treatment groups was observed (both *P* ‘s ≤ 0.025). The coloured dots represent the data points and the shaded areas surrounding the linear regression lines represent the 95% confidence intervals. Data distribution curves for both treatments is visualized on the right-hand side.

Baseline (day 0: 908 ± 52 [μg mL− ^1^]) protein content was significantly lower when compared to control and pollen-feed bees (*glm*; *z* = 3.68, *P* ‘s ≤ 0.001). Both treatment and age had a significant positive effect on the protein content in the hypopharyngeal glands (*glm*; both *z*’s > 2.24, *P* ‘s ≤ 0.025) and a significant positive interaction was observed between the two terms (*glm*; *z* = 2.13, *P* = 0.033). This resulted in a positive linear correlation between age and protein content across both groups (Fig 4). Irrespective of the treatment, HPG protein content significantly increased over time (*bmct*; all *z’s* > 9.03, *P’s* < 0.001). In comparison to the baseline bees, HPG protein content increased in three days (1083 ± 41 [μg mL− ^1^]), 10 days (1,542 ± 30 [μg mL− ^1^]) and 17 days old (1988 ± 95 [μg mL− ^1^]) bees by 19%, 70% and 118%, respectively. With respect to treatments, no significant difference in HPG protein content was observed on days three and 10 (*bmct*, both *z* > 1.71, both *P*’ > 0.38). However, a significant difference in HPG protein content was observed on day 17 (*bmct*, *z* = 5.25, *P* < 0.001); resulting in a 20% increase in protein content in pollen-fed (2,171 ± 104 [μg mL ^-1^]) compared to control (1,804 ± 26 [μg mL—^1^]) bees (Fig 4). This resulted in a HPG protein content increase on days three, 10 and 17 between pollen-fed and controls bees of 15%, 8% and 20%, respectively, where only the day 17 difference was significant.

## Discussion

Our data using *Tetragonula pagdeni* highlights the importance of an adequate pollen diet for stingless bee survival, HPG acini width and protein content, as well as suggests that temporal polyethism is the decisive factor governing HPG acini size. The findings are most likely explained by the beneficial effects of proteins and micro-nutrients for physiological development and health in bees. Further, the laboratory data confirm that HPG acini width and protein content are age-dependent, likely reflecting the age-specific tasks shown by social insect workers throughout their life. As expected, the HPG acini in nurses were the largest, reinforcing that the presence and attendance of brood triggers the development of this essential gland. As HPGs are key for collaborative brood care, and an essential element of eusociality [39], the presented data lay a foundation for future studies to investigate the impact of environmental stressors on a critical physiological trait in stingless bees–which may serve as a proxy to understand effects at the colony level.

Pollen consumption tends to occur shortly after the emergence of a bee and is most critical during the first few days of an individual’s life [1]. As we could not measure the consumption during our experiment, it is not possible to determine the daily pollen requirements of adult bees, nor can we elucidate potential correlations between pollen consumption and the measured physiological parameters. Nevertheless, the data underline the beneficial effects of pollen, complementing previous work in both adult solitary and social bees (e.g., [15, 40–44]. Pollen provides a wide range of proteins, lipids as well as macro- and micronutrients that are essential for optimal gland development, growth, and protein synthesis [16]. Indeed, pollen-deprived bees had smaller HPG acini as well as less protein content [45–48]. The benefits of a protein-rich diet were most evident when comparing the HPG protein content in the 17-day old pollen-fed laboratory bees with their respective controls which was increased by 20%. This increase likely corresponds with an improved ability to synthesise protein-rich and high in essential amino acid secretions by the secretory cells located in the HPGs [49]. Interestingly, in honey bees the quantity of HPG secretion was shown to positively correlate with acini size [24]. As we did not use the same bees to determine both HPG parameters we cannot confirm this finding for stingless bees. However, as the HPG acini width in 17-day old pollen-fed bees was only 3% larger than in the controls, the data suggest this may not be the case. Rather, our data appear to be in line with [25], wherein HPG acini size and protein content in the Eastern honey bee, *Apis cerana*, increased disproportionally with age.

An additional benefit of pollen consumption is the accumulation of storage proteins (e.g., vitellogenin) in the haemolymph [50]. Vitellogenin is known to have a positive effect on bee longevity [51]. This, in combination with well-developed organs and an improved gut microbiota [16, 52, 53], likely explains the enhanced survival observed in the pollen-fed bees. Our data underline that an adequate pollen diet is not only key for developing larva but also adult stingless bee survival. As foraging throughout the year can be reduced or even come to cease entirely due to resource scarcity or heavy rain (e.g., rainy seasons in Southeast Asia) [54], stingless bee colonies must ensure that they have sufficient pollen stores throughout the entire year. However, this may become increasingly difficult with projected shifts in seasonality and increasing extreme weather scenarios (e.g., extended dry/wet periods or fires) become far more likely to occur due to global climate change, especially in tropical regions such as Southeast Asia [55]. Therefore, a reduced or potential complete lack of forage, and the known negative effects of extended dry/wet periods on the production and nutritional composition of pollen [56], pose an additional stressor for wild and managed stingless bees [57]. Managed beekeepers could however already support their colonies by feeding nutritional supplements containing pollen to promote individual bee and colony health, similar to what is known for commercial honey bees [58].

While pollen is undisputedly an important factor for adequate physiological development in adult bees, our data reveal that age and temporal polyethism are more likely to be the underlying factors governing HPG development. Within 17 days, the hypopharyngeal gland acini width and protein content in pollen-deprived (i.e., control) bees drastically increased by 32% and 118%, respectively. Additional pollen feeding however only revealed a marginal positive effect on acini width (i.e., 3.5%) and protein content (i.e., 20%) after 17 days in comparison to the controls. Despite the distinct effect of pollen consumption observed for protein content, the age-dependent effect was far more pronounced. Our data suggest that the observed increased in measured HPG parameters is likely not dependent on pollen consumption but rather linked to inherent mechanisms associated with temporal polyethism. At the age of 13–18 days, stingless bees are commonly observed performing brood care tasks [4, 19, 59]. Our data support this as both acini width and protein content were the highest in laboratory bees on day 17. Interestingly, while the HPG acini size increased the most during days 10 and 17, the increase in protein content was most prominent between days three and 10. While only speculative, it appears plausible that in the absence of brood, workers began to downregulate the synthesis of costly proteins before reaching the typical nurse age. Possibly, as a response to save energy and resources that may better be reallocated to other physiological traits such as ovary development [60]. In contrast, acini size may be an inherent trait that cannot be decoupled by the absence of brood as we did not observe a decrease in size over time. However, additional studies are required to confirm these claims.

A limitation to our study is that the nurse and forager workers used in our experiment were not of a known age-cohort. This could have been achieved by using a mark-release-recapture method (e.g., [19]), allowing the collection of individuals of the same age as in our laboratory study. Nevertheless, we are convinced that the bees obtained from the colonies were indeed nurse and forager bees, as the acini size measured in our study lie well within the range of previous studies using stingless bees [17, 18]. Indeed, the HPG acini width in nurses was almost twice that of pollen-fed laboratory bees. This drastic increase in size is most likely attributed to the presence of brood [61] and the known positive correlation between gland activity and larval feeding [12, 25]. As expected, the HPG acini in forager bees was smaller than in nurse bees which corresponds with the glands no longer having to produce food for larvae [25]. Additional explanations for the size difference between laboratory and colony bees may be the source of pollen diet as well as variations in gut microbiota. Laboratory bees had a monofloral pollen source (*M*. *pudica*) and sucrose solution, whereas the colony fed bees likely had a polyfloral pollen diet [62] as well as access to honey—both known to positively affect tissue and organ development in bees [16, 63, 64]. Studies testing the effects of varying quality of pollen on the development of stingless bee physiology are scarce, and HPG acini width appears to be an ideal proxy for future studies investigating the effects of mono- vs polyfloral diets. In addition, pollen stored in stingless bee hives undergoes fermentation which alters the digestibility [65] and chemical composition which may have nutritional benefits for adults [66]. Also, fermented stored pollen is known to have a higher abundance and diversity of microorganisms (i.e., probiotics) that are known to be beneficial for workers and colony health [67], which may explain why adults prefer consuming stored older pollen over freshly collected pollen [68]. Given the provided pollen in our laboratory study was neither freshly collected nor fermented, and most certainly had reduced levels of microorganisms, this may explain the observed reduced HPG acini size development. Further, an optimal gut microbiota is essential for digesting nutrients [69] and can promote physiological development [70]. However, considering that adult bees acquire and develop their gut microbiota via contact with hive products (e.g., wax, or stored pollen and honey) as well as through trophallactic interactions with older bees [71], the laboratory bees likely had impaired digestive abilities which may have compromised their physiological development. Lastly, using the mark-release-recapture method and HPG acini width or protein content could enable to pinpoint exact transition phase of age-polyethism across different stingless bee species, as well as shed light on the average age of bees and how stochastic effects or environmental stressors may affect temporal polyethism.

## Conclusion

In summary, our data confirm the beneficial effects of pollen for adult bees and highlight that age and brood attendance govern HPG physiology. Such baseline data are key to improve our understanding of how bees and other insects may respond to environmental stress (e.g., pesticides and/or climate change). Additional baseline data on the rates of nectar and pollen consumption by larvae and adult stingless bees are urgently required to better understand the nutritional needs of these bees, but also to enable accurate predictions of the potential risk of pesticide exposure via contaminated food. Due to restrictions in experimental resources, quantification of the protein content in nurses and foragers was not feasible. Such data would enable to further unravel a potential correlation between HPG acini size and protein content beyond honey bees. The here presented methods will enable future studies to conduct comparative studies between stingless bees and honey bees under both laboratory and colony conditions. Further, it would also be interesting to investigated whether the HPGs of forager bees can be reactivated and display flexible secretory activity—as triggered in honey bees due to shifts in colony demographics, requeening or season changes [72]. Considering the integral role HPGs play in collaborative brood care and ultimately colony fitness, they seem to be an ideal sublethal fitness parameter for future toxicological studies [73]. This is particularly true, as past studies have revealed the negative effects of pesticides via contaminated sucrose on the development of HPGs in social bees [74–76]. In addition, as beebread (i.e., pollen stored in colonies) is often found to be contaminated with various pesticides [77], additional data on the pollen requirements of adult stingless bees would allow a more precise evaluation of the risk of pesticide exposure and their potentially hazardous effects. Lastly, recent data for Thailand project large declines in plant diversity due to climate change [78], which will reinforce the risk of malnutrition due to the reduced quality or even loss of forage. Thus, further research on the role of nutrition and climate change at the individual and colony level are required to put sustainable evidence-based mitigation measures in action to safeguard managed and wild stingless bees.

## Supporting information

S1 File (DOCX)
