# Supplementary material for: Age-dependent hypopharyngeal gland size and protein content of stingless bee workers, Tetragonula pagdeni
Source: PLoS One. 2024 Aug 16;19(8):e0308950. doi: 10.1371/journal.pone.0308950 (PMC11329107; doi:10.1371/journal.pone.0308950)
Supplement: S1 File — (DOCX) [file pone.0308950.s001.docx]

**Title:** Age-dependent hypopharyngeal gland size and protein content of stingless bee workers, *Tetragonula pagdeni*.

**Authors:** Lars Straub^1,2,3^, Tanatip Sittisorn^2^, Jinatchaya Butdee^2^, Woranika Promsart^2^, Athitta Rueangwong^2^, Domenic Camenzind^1^, Jakkrawut Maitip^2^

**Affiliations:**

^1^ Institute of Bee Health, Vetsuisse Faculty, University of Bern, Bern, Switzerland

^2^ Faculty of Science, Energy and Environment, King Mongkut's University of Technology North Bangkok, Rayong Campus, Rayong, Thailand

^3^ Centre for Ecology, Evolution, and Behaviour, Department of Biological Sciences, Royal Holloway University of London, Egham, United Kingdom

***Corresponding author:** [jakkrawut.m@sciee.kmutnb.ac.th](mailto:jakkrawut.m@sciee.kmutnb.ac.th)
**ORCID**: Jakkrawut Maitip - <https://orcid.org/0000-0001-6265-1421>

**Key words:** nutrition, physiology, polyethism, stingless bees, survival

**Table 1: Summary of sample sizes, obtained means, standard deviations (S.D.) and standard error for hypopharyngeal acini width at different time points and experimental conditions (i.e., laboratory or field).** Samples were taken from laboratory bees from either baseline, control, and pollen-fed treatment groups as well as for nurse and forager bees sampled from field colonies.

**Table 2: Summary of sample sizes, obtained means, standard deviations (S.D.) and standard error for hypopharyngeal acini width at four different time points (i.e., 0, 3, 10 and 17 days).**
